# Exploring social impairment in those with opioid use disorder: linking impulsivity, childhood trauma, and the prefrontal cortex

**DOI:** 10.1186/s12888-025-06503-1

**Published:** 2025-03-03

**Authors:** Thais Costa Macedo de Arruda, Laura Sinko, Paul Regier, Altona Tufanoglu, Adrian Curtin, Anne M. Teitelman, Hasan Ayaz, Peter F. Cronholm, Anna Rose Childress

**Affiliations:** 1 Psychology and Neuroscience Department, College of Liberal Arts, Temple University, Philadelphia, PA USA; 2 Nursing Department, College of Public Health, Temple University, Philadelphia, PA USA; 3 Perelman School of Medicine, University of Pennsylvania, Philadelphia, PA USA; 4 School of Biomedical Engineering, Science and Health Systems, Drexel University, Philadelphia, PA USA; 5 College of Nursing, School of Nursing, Thomas Jefferson University, University of Pennsylvania, Philadelphia, PA USA; 6 Department of Psychological and Brain Sciences, College of Arts and Sciences, Drexel University, Philadelphia, PA USA; 7 A.J. Drexel Autism Institute, Drexel University, Philadelphia, PA USA; 8 Drexel Solutions Institute, Drexel University, Philadelphia, PA USA; 9 Center for Injury Research and Prevention, Children’s Hospital of Philadelphia, Philadelphia, PA USA; 10 Department of Family Medicine and Community Health, Center for Public Health, Leonard Davis Institute of Health Economics, University of Pennsylvania, Philadelphia, PA USA

**Keywords:** Opioid use disorder, Social functioning, Impulsivity, Childhood trauma, Prefrontal cortex, Brain, Behavior

## Abstract

**Background:**

Challenges with social functioning, which is a hallmark of opioid use disorder (OUD), are a drawback in treatment adherence and maintenance. Yet, little research has explored the underlying mechanisms of this impairment. Impulsivity and corresponding neural alterations may be at the center of this issue. Childhood adversity, which has been linked to both impulsivity and poorer treatment outcomes, could also affect this relationship. This study explores the relationship between impulsivity, social functioning, and their neural correlates in the prefrontal cortex, while examining the potential moderating effects of childhood trauma in individuals recovering from OUD.

**Methods:**

Participants with (*N* = 16) and without (*N* = 19) social impairment completed a survey (e.g., social functioning, Barrat’s Impulsivity Scale, Adverse Childhood Experiences (ACEs) and cognitive tasks while undergoing neuroimaging. Functional near infrared spectroscopy (fNIRS), a modern, portable, wearable and low-cost neuroimaging technology, was used to measure prefrontal cortex activity during a behavioral inhibition task (Go/No-Go task).

**Results:**

Those who social functioning survey scores indicated social impairment (*n* = 16) scored significantly higher on impulsivity scale (t [33]= -3.4, *p* < 0.01) and reported more depressive symptoms (t [33] = -2.8, *p* < 0.01) than those reporting no social impairment (*n* = 19). Social functioning was negatively correlated with impulsivity (*r*=-0.7, *p* < 0.001), such that increased impulsivity corresponded to decreased social functioning. Childhood trauma emerged as a moderator of this relationship, but only when controlling for the effects of depression, B=-0.11, *p* = 0.023. Although both groups had comparable Go/No-Go task performance, the socially impaired group displayed greater activation in the dorsolateral (F(1,100.8) = 7.89, *p* < 0.01), ventrolateral (F(1,88.8) = 7.33, *p* < 0.01), and ventromedial (F(1,95.6) = 7.56, *p* < 0.01) prefrontal cortex duringthe behavioral inhibition task.

**Conclusion:**

In addition to being more impulsive, individuals with social impairment exhibited greater activation in the prefrontal cortex during the Go/No-Go task. Furthermore, the impact of impulsivity on social functioning varies depending on ACEs, such that higher levels of ACEs corresponded to a stronger negative relationship between impulsivity and social functioning, highlighting its importance in treatment approaches. These findings have implications for addressing social needs and impulsivity of those in recovery, highlighting the importance of a more personalized, integrative, and trauma-informed approach to intervention.

## Background

**Opioid use disorder (OUD)** is a significant public health issue characterized by the compulsive consumption of opioids, which are substances (such as heroin, morphine, codeine, and fentanyl) associated with pain relief. Worldwide, there were approximately 600,000 deaths related to drug use in 2019, with almost 80% of them associated with opioids [1]. In the United States, there has been a drastic increase in overdoses since 2019, coinciding with the COVID-19 pandemic, which resulted in nearly 110,000 reported overdose deaths by the end of 2022 [1], largely driven by opioids (~ 72%). This recent surge in overdoses calls for an increase in supporting recovery and improving treatment. Studies have shown that patient-provider relationships and support networks are fundamental in treatment adherence, maintenance, and satisfaction [2]. However, patients in recovery often exhibit challenges in developing and maintaining these relationships, which can represent a drawback. To provide more effective care, it is fundamental to understand the underlying mechanisms behind the social challenges that patients in recovery might face.

**Social functioning** is defined as the ability to participate in social roles and activities, and it is often affected by the chronic use of opioids [3, 4]. Challenges with or related to social functioning are key criteria in the diagnosis of OUD (DSM-5). Social functioning encompasses many skills, such as empathy, communication, adaptability, and self-regulation. These allow individuals to navigate interpersonal interactions, detect social cues and emotions, and ultimately develop healthy relationships. A case-control study has shown thatindividuals with OUD exhibit notable deficits in emotional perception and social inference compared to those without OUD, underscoring significant impairments in these crucial social abilities [5]. Social Cognitive Theory also explores the significance of self-regulatory mechanisms, underscoring that the ability to manage one’s emotions, thoughts, and behaviors is crucial for during social interactions [6]. In line with this idea, previous work has suggested that inhibitory control might be essential to social functioning [7]. If controlling impulses is crucial to successfully participating in society, impulsivity may be central to OUD patients’ social challenges.

**Opioid use disorder (OUD) and impulsivity.** One of the critical factors associated with OUD is impulsivity. Individuals who are impulsive exhibit difficulties with self-regulation and higher risk-taking behaviors [8, 9], seeking pleasure and reward while overlooking possible consequences. Impulsivity exacerbates drug-seeking behaviors, which may lead to the development of OUD. Indeed, previous studies have found that impulsivity is a risk factor for opioid misuse as well as the development of OUD [10, 11]. From a neurocognitive perspective, individuals with more impulsivity may also exhibit difficulties with “disinhibition”, referring to the top-down control of more reflexive (i.e., subcortical, or bottom-up) responses [12]. The prefrontal cortex (PFC), an area often associated with the inhibition of responses, is known to be affected by the chronic use of opioids. Studies have shown that OUD is associated with disrupted activity in the PFC [13, 14], which may lead to impairments in inhibitory control. In heroin-dependent patients, a study found that impulsivity was correlated with a decrease in gray matter volume in the PFC [15]. Notably, research has consistently shown differential activation of the PFC for heroin users compared to non-users during inhibition tasks [16, 17]. Hence, impulsivity may be associated with disrupted inhibition and contribute to the development and maintenance of OUD. OUD can lead to alterations in the structure and function of the PFC, exacerbating impulsiveness and drug-use behaviors.

The above literature suggests impulsivity is a risk factor for the development and maintenance of OUD (i.e., more impulsivity increases the probability of OUD-related outcomes), there are few studies investigating how it further impacts individuals during recovery. Lack of impulse control in social interactions, disruption of social rules, and inability to regulate emotional responses can all hinder one’s ability to interact socially. Hence, self-regulation represents a critical factor in the control of social behavior [18]. Consistently, on the neural level, lesions to the PFC have been associated with social impairment [19–21]. Moreover, disruptions in the PFC circuitry have been observed in patients with psychiatric disorders commonly associated with deficits in social functioning, such as schizophrenia and autism spectrum disorder [22]. Therefore, the underlying dysregulation of PFC circuitry may affect one’s ability to self-regulate, leading to challenges in social situations. Overall, underlying mechanisms in the PFC may contribute to impulsive behaviors that impact social functioning during treatment.

**Adverse Childhood Experiences.** Exposure to adversity during childhood, including physical, sexual, and emotional abuse, as well as neglect, has also been linked to OUD. Among individuals diagnosed with OUD, approximately 41% of women and 16% of men are estimated to have a history of childhood sexual abuse [23]. Since childhood is a pivotal time in development, experiencing trauma during this period can have long-lasting effects that encompass physical health, behaviors, and neurobiology. For example, studies have shown that childhood adversity is linked to both functional and structural alterations in the brain [24]. On a behavioral level, those with a history of childhood trauma are more likely to seek drugs as a coping mechanism to deal with the emotional burden [25]. Adversity during childhood has also been associated with both impulse control [26, 27] and interpersonal [28] challenges in adulthood. For those with OUD, in particular, childhood trauma has been linked to poorer treatment outcomes [23]. This connection may be explained by the heightened social challenges faced by those with comorbid OUD and a history of childhood trauma. Social functioning may be an underexplored, yet promising, area to improve outcomes for those with OUD [29, 30]. However, it remains unexplored whether adverse childhood experiences impact the relationship between impulsivity and social functioning in individuals recovering from OUD.

**This study** sought to understand what lies behind the social challenges patients face in recovery. First, it investigated if individuals with impaired social functioning are more impulsive [1]. Next, it exploredwhether activation of the dorsolateral PFC during an impulse control task differs in those with poor social functioning [2]. Finally, it analyzed whether childhood trauma moderates these relationships at a behavioral level [3]. We hypothesized that those with social impairments would be more impulsive [1] and display significantly greater activity in the dorsolateral PFC during a novel version of the Go-NoGo task [2] than those without social impairment. We further hypothesized that childhood trauma would moderate the relationship between impulsivity and social functioning, such that those with greater traumatic experiences would display more impulsivity and increased challenges in social functioning [3]. Understanding how past experiences may shape behavior during recovery can help tailor interventions to improve treatment adherence. These intricate and highly individualized experiences may be a gateway to maximize chances of recovery and later social reintegration.

## Methods

This is a secondary analysis using data from two pilot studies, examining the impact of opioid use and sexual violence on executive functioning. Thirty-five participants (women, *n* = 29) were recruited for this study. The present study analyzed the survey, neuroimaging, and behavioral task data.

### Recruitment

Individuals were recruited through community organizations that support those on a medication-assisted treatment for OUD in a large metropolitan area, using flyers, email listservs, and presentations at in-person programmatic events. Interested people reached out through phone or email to be screened for our study. Participants were eligible to participate in the study if they were [1] between the ages of 18–60 [2], on a medication-assisted treatment for OUD [3], able to attend an in-person brain imaging session [4], not pregnant, and [5] not presenting with schizophrenia or bipolar with current manic state. All participants offered informed consent before participation, and the University of Pennsylvania Institutional Review Board approved all procedures in this study.

### Procedure

The study was divided into two visits. During the first visit, which lasted around 2 h, participants completed an online survey and the Penn Computerized Neurocognitive Battery [31] tasks. They then received a resource list and were asked to schedule a second two-hour visit. In the second visit, participants performed another series of cognitive tasks while connected to a non-invasive brain monitoring device called functional near-infrared spectroscopy (see section on *fNIRs*). Individuals were compensated for their time.

### Survey measures

*Demographics* included age, zip code, gender, race, ethnicity, and socioeconomic measures such as the highest education level, mother’s education level, current employment, and income.

*Social functioning* was assessed using the PROMIS Ability to Participate in Social Roles and Activities [32]. This study used the short form with eight questions evaluating the perceived ability to participate in social activities (e.g., “I have trouble doing all of my regular leisure activities with others”). Items were reversed scored, so higher scores represented fewer perceived limitations and, hence, higher social functioning. Using the PROMIS scoring system [33], the final scale score was transformed into a t-score with a mean of 50 and a standard deviation 10. The t-score metric allows us to compare the participants’ scores to the US general population, with scores below one standard deviation of the mean representing some impairment. T-scores are further subdivided into mild (45 − 40), moderate (40 − 30), and severe (below 30) impairment. We used both a summed score (continuous variable) and a categorical variable for analysis purposes. Those with social functioning levels one standard deviation below the general population average (equivalent to a t-score of 40) were categorized as part of the socially impaired (SI) group. At the same time, those with scores of 40 or above were included in the not socially impaired (N-SI) group.

*Impulsivity* was assessed with Baratt’s Impulsiveness Scale (BIS) [34], a self-report measure composed of thirty questions (e.g., “I do things without thinking”). It uses a 4-item metric that ranges from “rarely/never” to “almost always/always,” with higher scores representing greater impulsivity. The BIS has been previously used and validated in different populations, with an alpha coefficient of around 0.69 to 0.83 [35].

*Adverse Childhood Experiences* were measured using the Philadelphia Adverse Childhood Experiences (PHL ACEs) survey [36]. This is an expanded version of the conventional Adverse Childhood Experiences (ACE) containing 16 items used to assess exposure to abuse and trauma during childhood. This version is especially useful for certain sociodemographic groups, including people of color and low-income backgrounds, as it incorporates questions sensitive to those groups’ experiences [36]. The expanded version includes potential community-level adversity, assessing exposure to racism, bullying, and neighborhood violence. Following the conventional ACEs scoring sheet, a total sum score was generated, with greater scores representing greater exposure to childhood adversity. Although previous studies have revealed that significant health outcomes begin to appear with scores of 4 or higher [37], this approach disregards the variances and nuances that might arise from exposure to higher levels of adversity. Considering the increased levels of childhood trauma observed in our sample (see description in the *Results* section), we chose to use a cumulative score approach to encompass these variations. This method has been previously used and replicated in many studies [38].

*Depression* was assessed using the Quick Inventory of Depressive Symptomology (QIDS). The QIDS is a 16-item scale highly aligned with the DSM-V criteria and used to measure depressive signs and symptoms, with a focus on emotional distress [39]. Each question was scored on a 0–3 severity scale, and the total score was calculated by adding up all the responses in a range of 0 to 48 - higher scores meaning higher depressive symptoms. The QIDS has been demonstrated to be both reliable (a = 0.80 to 0.94) [40] and strongly correlated to other validated measures such as the Beck Depression Inventory and the Hamilton Rating Scale for Depression [40].

### Neuroimaging data collection

*Functional near-infrared spectroscopy (fNIRS)* is a wearable neurotechnology used to measure changes in cortical oxygenation changes using near infrared light with non-invasive wearable sensors over the scalp [41].Traditionally, research involving populations in recovery or with a history of trauma exposure has often relied on conventional devices such as functional magnetic resonance imaging (fMRI). In this study, we chose to use fNIRS for three main reasons: portability, affordability, and comfort. Contrary to conventional technologies, fNIRS is a small and lightweight device, allowing the data collection to be carried out in a variety of environments such as clinics, hospitals, and the field [42, 43], which reduces location restrictions and increase accessibility for participants. Following the size discrepancy, fNIRS also has a lower cost when compared to other traditional devices [43], reducing the cost of the overall research process.

Moreover, in contrast to the discomfort posed by fMRI, fNIRS has a simplified head-band structure, which prevents participants from undergoing long periods of stillness in confinement and enduring loud noises. This may help reduce patients’ anxiety and distress, minimize potential triggers, and increase comfort during data collection [42–45]. Hence, fNIRS emerges as a technique that is sensitive to the unique needs and challenges of individuals with exposure to trauma and in recovery from opioid use. Aside from being affordable, this is a more trauma-informed and community-based technology, helping foster a more accommodating research environment and making it ideal for our study.

For neural data collection, participants sat in a room with a computer and a wearable fNIRS sensor Model 1200 (fNIR Devices LLC) system with a flat sensor pad placed over the anterior PFC and secured with elastic fabric. While completing a task (described below), activity in the PFC was measured using four light sources and ten detectors resulting in 16 optodes (cortical measurement areas) as described before [46]. There are two light wavelengths (730 and 850 nm) with a temporal resolution of 500 milliseconds. The arrangement of the light source and the detectors on the device resulted in 16 active optodes, or channels [46] distributed from dorsal to ventral and lateral to medial brain.

**Fig. 1:**
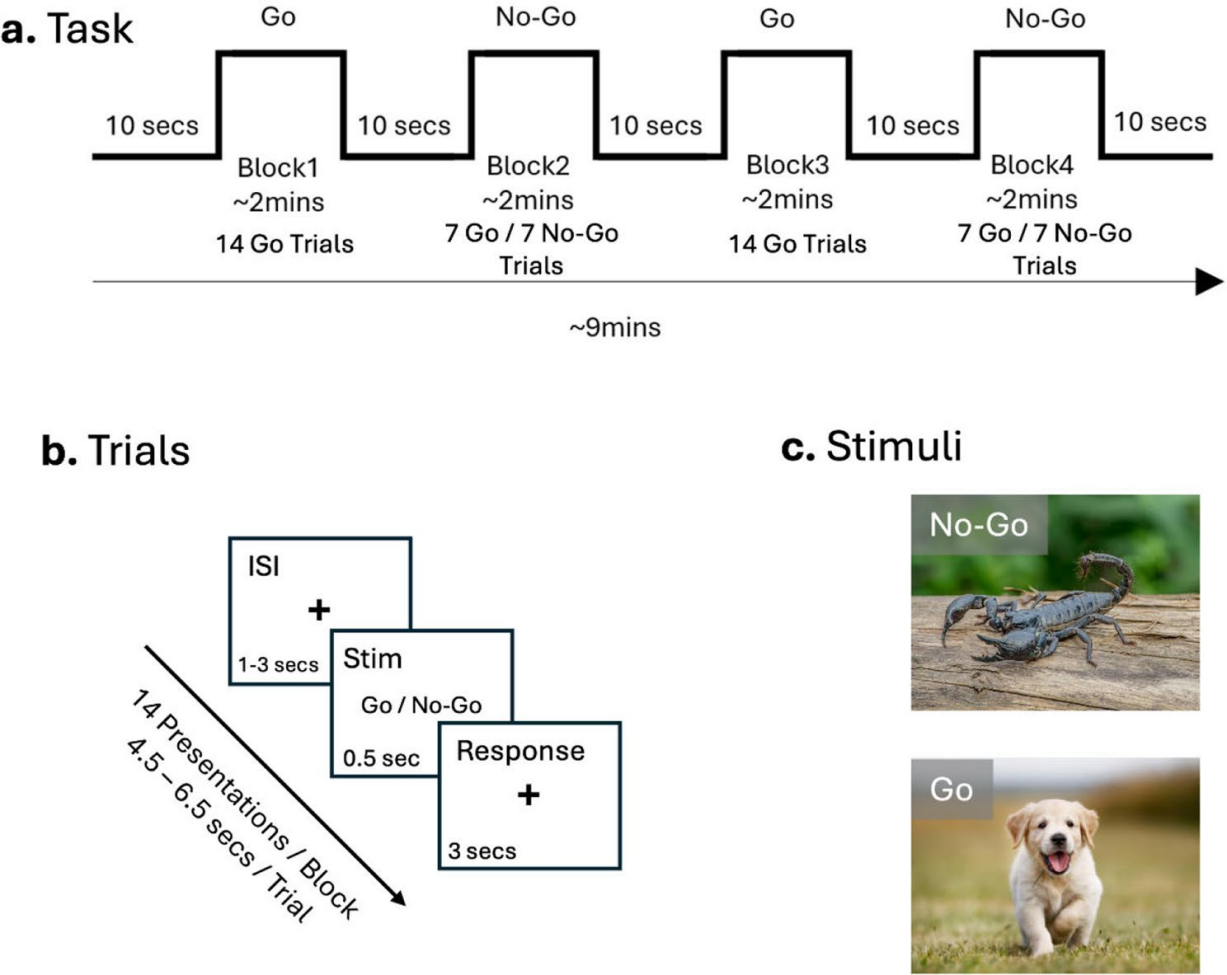
Affective Go/No-Go Task. (**a**) Design of task with alternating Go and No-Go blocks, with 1.5–2 min per block and 10 s in between each block. For Go blocks, there were 14 Go trials, and for No-Go blocks, there were 7 Go and 7 No-Go trials. (**b**) Trials consisted of an ISI 1–3 s in duration, followed by a 0.5 s presentation of either Go or No-Go stimuli, and then 3 s for a response. (**c**) Example images of Go (puppies) and No-Go (scorpions) stimuli

*The fNIRS task* used was an affective version of the well-known Go-No-Go task, also called the Spiders-No, Puppies-Go task [47] on a computer with a 15.6-inch monitor using PsychoPy^®^ software [48]. This affective task uses appealing (puppies) and aversive (scorpions) images to increase the ecological validity of the traditional task. Instead of the abstract stimuli typically presented (i.e., letters and numbers), using evocative images makes the task more engaging [47]. This is particularly important in this study as we use it to observe neural activation; hence, approach/avoidance stimuli can help simulate responses more similar to environmental conditions (See Fig. 1).

As in the typical task, participants received instructions and completed practice trials before commencing the task. They were instructed to press the space bar when shown ‘Go’ stimuli and refrain from pressing when shown ‘No-Go’ stimuli. The task consisted of four blocks, comprising two ‘Go’ and two ‘No-Go’ conditions, in which subjects were shown stimuli for 0.5s and had 3 s to respond. This was followed by an inter-stimulus interval (ISI)– fixation cross appearing on the screen– for a random interval ranging from 1, 2–3 s. Each block consisted of 14 images, with an inter-block interval of approximately 10 s. The Go trial is used as a form of control, as only appealing photos are shown (100% Go stimuli), while in the No-Go trial, both aversive (50%, *n* = 7) and appealing (50%, *n* = 7) images are shown, enabling the assessment of impulse control abilities.

### Analysis

*Signal processing.* For each participant, raw light intensity data (wavelengths of 730 nm and 850 nm) were continuously sampled from 16 anterior PFC regions at a rate of 2 Hz. The preprocessing pipeline included low-pass filtering (FIR with a linear phase) at 0.1 Hz to eliminate high-frequency noise and physiological artifacts such as cardiac and respiration cycles. fNIRS data for each block were extracted using time synchronization markers received through a serial port during the experiment. Subsequently, the acquired data was analyzed to calculate alterations in oxygenated hemoglobin (HbO) concentrations using the Modified Beer-Lambert Law. Motion artifacts were corrected by applying Temporal Derivative Distribution Repair as described by Fishburn et al. (2019) [49]. The hemodynamic response at each optode was averaged across time for each trial block to provide a mean hemodynamic response at each optode for each block. Relative changes in oxyhemoglobin (HbO) and deoxyhemoglobin (HbR) for each activation condition were calculated relative to distinct local baselines measured during the first ten samples at the beginning of the Go and NoGo blocks. Changes in oxyhemoglobin and deoxyhemoglobin for the two activation conditions were calculated relative to respective local baseline segments.

*Statistical Analyses* were conducted using IBM SPSS Statistics (Version 27) and RStudio. The significance criterion was set to *a* = 0.05. We used descriptive statistics, including mean and standard deviations, to describe sociodemographic characteristics. To understand group differences (socially impaired versus not) of depression (QIDS and impulsivity (BIS) scores, two-sample t-tests were conducted. We also used Pearson’s correlations to examine potential linear relationships between impulsivity (BIS) and social functioning (total [PROMIS-Social Functioning] score). To explore possible moderation effects of childhood trauma, we fitted a linear model (estimated using ordinary least squares) to predict this interaction. Variables significantly related to our dependent or independent variables were included as covariates in our model to control for confounds.

Considering depression is both commonly comorbid with OUD [50] as well as a consistent outcome of childhood trauma experiences [51], it seemed crucial to control for the potential effects of this variable. In line with previous literature [52], individuals in our sample with social functioning challenges displayed greater levels of depressive symptoms. Therefore, we decided to look beyond the depression symptom burden by controlling for potential variations in our moderation analysis.

In addition, we analyzed the Affective Go/No-Go task at both behavioral and brain activation levels. Analysis of variances (ANOVAs) was used to compare group performance differences, examining response time and accuracy (correct responses per trial). We used linear mixed effects model analysis for neuroimaging data to estimate the main effects of group (socially impaired vs. not) vs. conditions (Go vs. No-Go trials) and their interaction. This model was chosen because it handles repeated measures more effectively than ANOVAs. Our task presented repeated data points for the same trial type, and using this analysis enabled the data within each group to be treated as related instead of independent. The dependent variable was HbO (used as a proxy for brain activation) at each optode, from one to sixteen. In this context of multiple comparisons, False Discovery Rate (FDR) corrections were used to control for the inflation of positive results.

## Results

### Sample

Thirty-five men and women affected by substance use were recruited for this study. Of those, 32 self-described as having an addiction or substance use disorder and were on medication-assisted treatment for OUD, and 29 shared that they used opioids in the past year. The inclusion of individuals who did not self-report an addiction or disorder was based on the understanding that their perspectives can contribute valuable insights into the experiences of individuals affected by substance use, regardless of self-reported status. According to the PROMIS Ability to Participate in Social Roles and Activities t-score system, 16 participants had some social impairment, ranging from mild (*n* = 8) to moderate and severe (*n* = 8). Participants experienced a considerable degree of adversity during childhood, with only two individuals reporting no exposure to adverse childhood experiences (ACEs). The overall sample displayed an average cumulative score of 5.89 on the PHL ACES scale. Breaking it down into quartiles, 7 participants reported 1–3 ACEs, 13 reported 4–6, 6 reported 7–8, and 7 reported over eight adverse experiences. For more information, see Table 1.

**Table 1:** Demographic and clinical characteristics of the sample with statistical comparison between SI-Y and SI-N subgroups

Variable	Total Sample (*n* = 35)	SI-Y (*n* = 16)	SI-*N* (*n* = 19)	Statistics
Age (Mean)	46.5	45.3	47.6	t = 0.76 (*p* = 0.45)
Sex At Birth (% Female)	80%	100%	63%	c2 = 7.4 (*p* = 0.007)**
Race (% Black) (*n* = 34)	56%	33%	72%	c2 = 4.4 (*p* = 0.04) *
Hispanic Y/N (*n* = 34)	6%	6%	6%	c2 = 0.01 (*p* = 0.73)
Education (Self, %) (*n* = 34)				c2 = 1.83 (*p* = 0.4)
Did not finish HS HS or GED Some college or more	15% 41% 44%	13% 31% 56%	17% 50% 33%	
Education (Mother, %) (*n* = 34)				c2 = 4.7 (*p* = 0.1)
Did not finish HS HS or GED Some college or more	21% 41% 38%	6% 56% 38%	33% 28% 39%	
Self-reported Substance Use Disorder (% Yes) (*n* = 33)	94%	94%	94%	c2 = 0.00 (*p* = 0.97)
Drug Use in Past Year (*n* = 33)				
Opioids	88%	81%	94%	c2 = 1.3 (*p* = 0.28)
Stimulants	45%	44%	47%	c2 = 0.04 (*p* = 0.85)
Cannabis	36%	31%	41%	c2 = 0.35 (*p* = 0.55)
Alcohol	45%	56%	36%	c2 = 1.5 (*p* = 0.23)
Self-reported Mental Health Disorder (% Yes)(*n* = 33)	79%	94%	65%	c2 = 4.2 (*p* = 0.05)
Impulsivity (Mean)	73.2	80.2	67.3	t = -3.4 (*p* = 0.002)**
Depression (Mean)	15.8	19.3	12.9	t = -2.8 (*p* = 0.01)**
Adverse Childhood Experiences (Mean)	5.9	7.3	4.7	t = -2.1 (*p* = 0.04)*

**p < 0.01, * *p* < 0.05

### Survey results

#### Is social impairment associated with higher impulsivity scores?

**Fig. 2:**
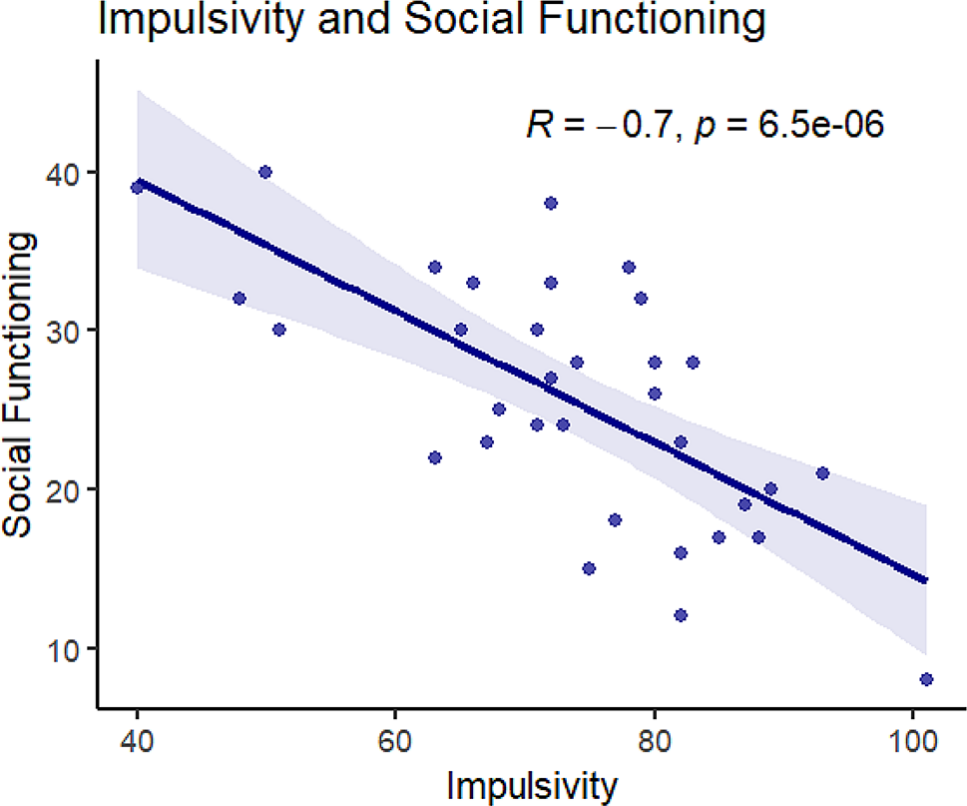
Scatter plot for the correlation between impulsivity and social functioning. Scatter plot illustrating the relationship between impulsivity and social functioningamong participants. The linear regression line represents the fitted relationship, with a shaded area indicating the confidence interval. The Pearson correlation coefficient indicates the strength and direction of the association

Impulsivity and social functioning were negatively correlated, such that increased impulsivity was associated with decreased social functioning (*r*=-0.7, *p* < 0.001; See Fig. 2). T-tests also revealed that the socially impaired group had significantly higher impulsivity scores (M = 80.2) than the group without social impairment (M = 67.3, t [33]= -3.4, *p* < 0.01; See Table 1).

#### Exploratory: do those with social impairment differ in depression levels compared to those without?

The socially impaired group reported significantly higher depressive symptom scores (M = 19.3) compared to those without (M = 12.9, t [33] = -2.8, *p* < 0.01; See Table 1).

#### Does childhood trauma moderate the relationship between impulsivity and social functioning when controlling for depression symptoms?

**Fig. 3:**
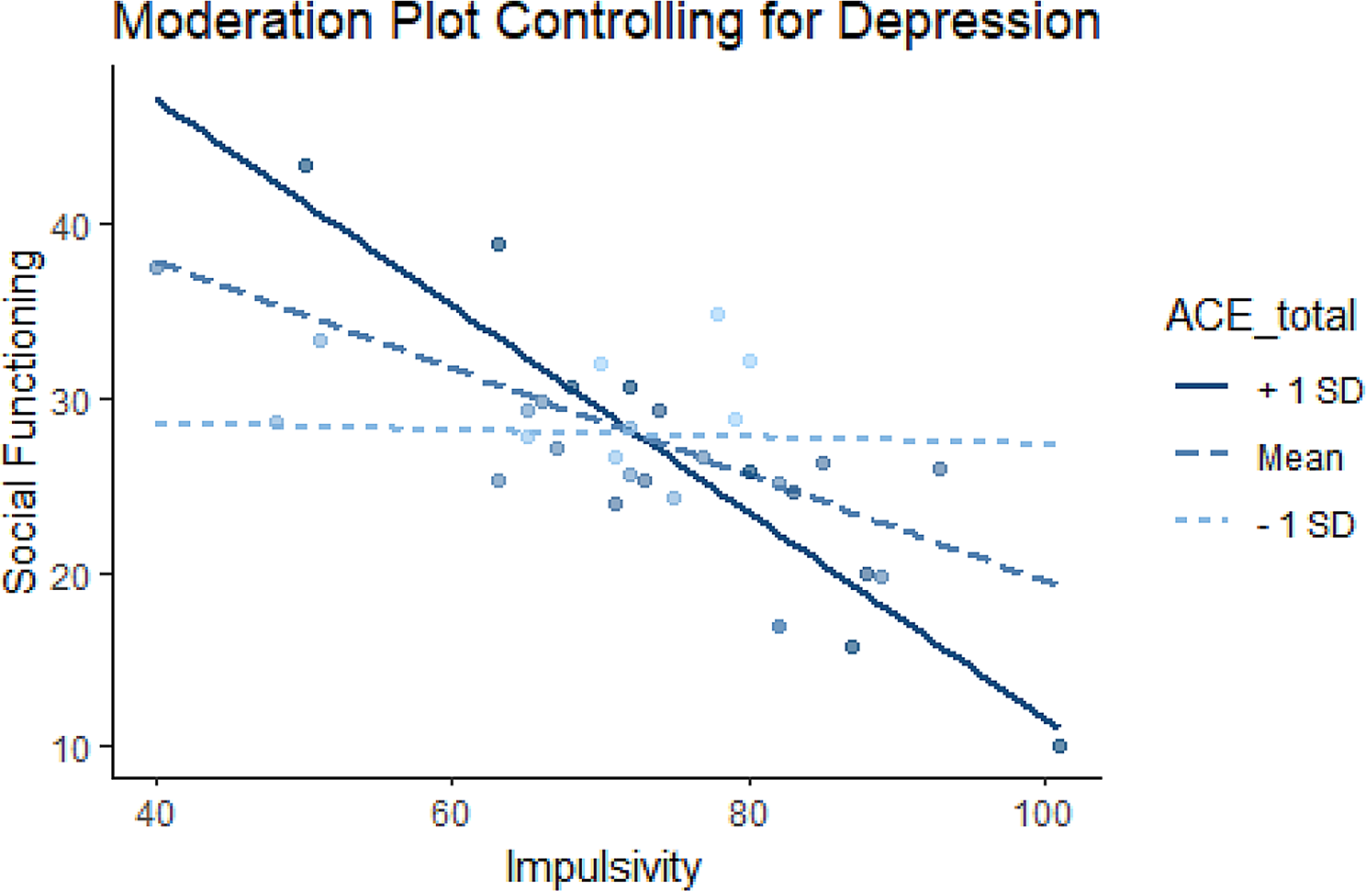
Moderation plot for ACEs, impulsivity, and social functioning, while controlling for depression symptoms. The solid line represents ACEs score one standard deviation above the mean, the dotted blue line represents ACEs score at the mean, and the light blue dotted line represents scores one standard deviation below the mean. There is a significant moderator effect of ACEs on the relationship between impulsivity and social functioning (B=-0.11, *p* = 0.023). Simple slopes analysis revealed that the relationship between social functioning and impulsivity was not significant for individuals with low ACEs (light blue dotted line) (b = -0.02, SE = 0.18, *p* = 0.92), but significant for those with high ACEs (solid bue line) (b = -0.59, SE = 0.16, *p* < 0.001)

We investigated whether the relationship between social functioning and impulsivity is moderated by exposure to childhood adversity, including depressive symptoms (using the QIDS) as a covariate. This yielded a significant moderator effect of ACEs on the relationship between impulsivity and social functioning (B=-0.11, *p* = 0.023). Additional simple slopes analysis revealed that, when looking at low ACES (one standard deviation below the mean), the relationship between social functioning and impulsivity was not significant (b = -0.02, SE = 0.18, *p* = 0.92). Conversely, when looking at high ACES (one standard deviation above the mean), the relationship was significant (b=-0.59, SE = 0.16, *p* < 0.001). This interaction remained significant for those with experiences of childhood adversity close to the mean (M = 5.11, B=-0.31, SE = 0.13, *p* < 0.05; See Fig. 3).

### Neuroimaging results

#### Are there differences in brain activation between the groups (socially impaired vs. not) when engaging in impulse control tasks?

**Fig. 4:**
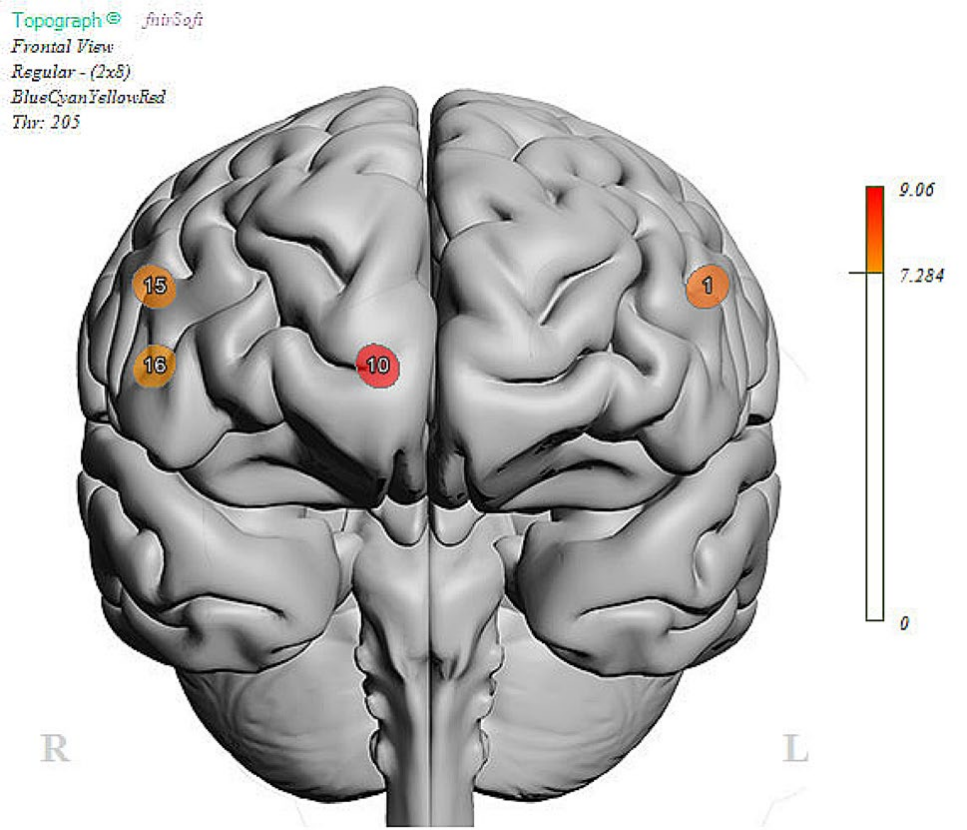
Parametric plot for main effects of Group (SI vs. N-SI) during affective Go-NoGo task. This parametric plot shows results from a linear mixed effects model, with corresponding F-score values representing significant (FDR-corrected) main effects of group at optode 1 (left dorsolateral PFC), optode 10 (right ventromedial PFC), optode 15 (right dorsolateral PFC), and optode 16 (right ventrolateral PFC)

**Fig. 5:**
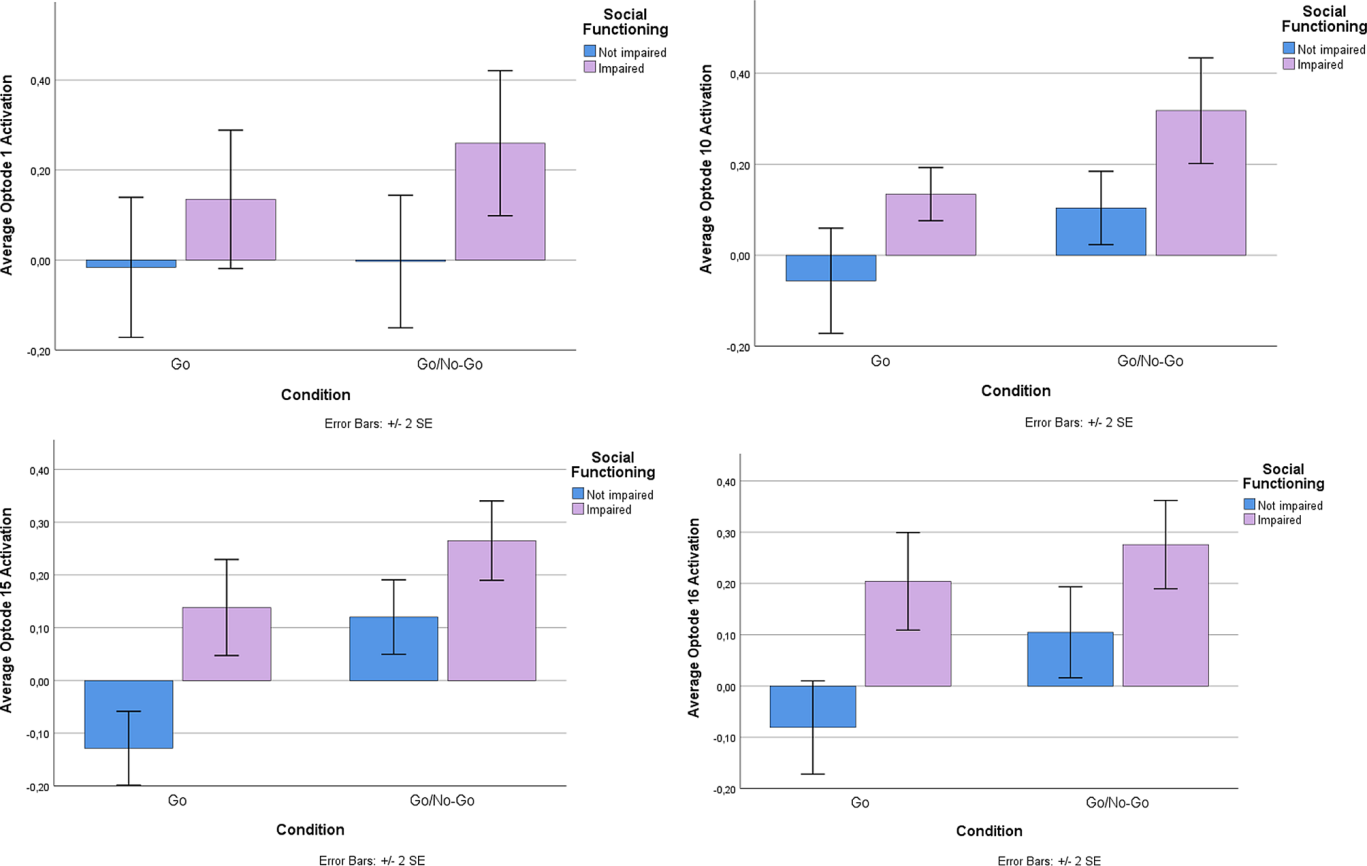
Bar graphs for average HbO activity from fNIRS Optodes that had a significant main effect of group (SI vs. SI-N). For each individual barplot, y-axis indicates average HbO changes in each optode. Blue bars represent the SI-N group while purple bars represent the SI group. On the left of each figure, HbO activity is shown for Go condition and on the right activity is shown for Go/No-Go. Whiskers represent the standard error of the mean (SEM)

Using the Go-No-Go task, a well-known and established task to measure abilities to control impulses, we examined performance brain activity in the PFC between those with and without social impairment. ANOVA analysis revealed no differences in performance (Correct Responses (Go): SI-*N* = 95%, SI-Y = 98%, ND; Correct Responses (No-Go): SI-*N* = 96%, SI-Y = 96%, ND). Despite equal performance on the task, linear mixed model analysis revealed activation differences at the level of the brain (Table 2). There was a main effect of group (FDR corrected) in Optode 1, the left dorsolateral PFC (F(1,100.8) = 7.89, *p* < 0.01), Optode 10, right ventromedial PFC (F(1,75.8) = 9.06, *p* < 0.01), Optode 15, right dorsolateral PFC (F(1,95.6) = 7.56, *p* < 0.01), and Optode 16, right ventrolateral PFC (F(1,88.8) = 7.33, *p* < 0.01; See Fig. 4). Estimated fixed effects suggest that the socially impaired group had an increased (primarily dorsolateral) cortical response during the task compared to the non-socially impaired group (see Fig. 5). The main effects of condition (Go vs. No-Go) were found only in Optode 10 (F(1,75.8) = 6.58, *P* = 0.012) and Optode 15 (F(1,95.6) = 6.23, *p* = 0.014). No interaction effect was observed between the condition and group.

**Table 2:** The main effect of Social Impairment for different optodes

Optode	F-value	Num DF	Denum DF	*P*-value
1	7.89	1	100.8	0.006**
2	1.79	1	99.7	0.184
3	0.22	1	91.7	0.642
4	4.20	1	99.1	0.043*
5	1.03	1	81.5	0.314
6	1.83	1	99.7	0.179
7	0.53	1	87.8	0.468
8	2.70	1	66.7	0.105
9	2.64	1	85.2	0.108
10	9.06	1	75.8	0.004**
11	2.15	1	88.2	0.146
12	0.000	1	93.0	0.997
13	2.16	1	97.5	0.145
14	3.46	1	82.5	0.067
15	7.56	1	95.6	0.007**
16	7.33	1	88.1	0.008**

*Significant (*p* < 0.05); **Significant (*p* < 0.01);

#### Exploratory: are there differences in brain activation between the groups (socially impaired vs. not) during impulse control tasks when controlling for depression?

Considering the previously stated prevalence of depression symptoms in this population, we decided to further control for depression in the neural analysis portion of our study. Results revealed that only two Optodes of interest remained significant when performing the same linear mixed models analysis with depression as a covariate: Optode 1, the left dorsolateral PFC(F(1,97.9) = 13.54, *p* < 0.001), and Optode 10, the right ventromedial PFC (F(1,75.32) = 7.42, *p* < 0.01), while the main effects of social impairment on Optode 15, right dorsolateral PFC(F(1,96.2) = 3.47, *p* = 0.066) and Optode 16, right ventrolateral PFC (F(1,87.8) = 3.60, *p* = 0.062) did not reach significance after controlling for depression symptoms. Previously observed main effects of task condition (Go vs. No-Go) remained significant in Optode 10 (F(1,74.9) = 6.47, *p* = 0.013) and in Optode 15 (F(1.,95.7) = 6.22, *p* = 0.014).

## Discussion

Identifying underlying mechanisms of social functioning challenges related to substance use is critical for developing more effective interventions. Our study found that patients in recovery from OUD who exhibit some level of social impairment have higher impulsivity scores, greater depressive symptoms, and increased PFC activation during both the Go and No-Go portions of an inhibitory task compared to those without any detectable social impairment. We also found that, when controlling for depressive symptoms, ACEs moderated the relationship of social impairment and impulsivity, such that those with a higher degree of trauma exposure displayed increased impulsivity and decreased social functioning. Despite the preliminary nature of this pilot study, these results suggest the importance of integrative care framed in trauma informed approaches, considering not only mental health symptom burden but also trauma history to help promote social functioning. This focus can facilitate the ability to build and form relationships which are critical for recovery.

Previous literature has found that impulsivity is both a risk factor and a contributor to the maintenance of OUD [53], with some highlighting that more impulsivity is linked to poorer treatment outcomes [54]. However, very few studies investigate what lies behind impulsivity and how it further impacts patients during recovery. One study traced negative indicators of social functioning in patients recovering from OUD [55], analyzing demographic (age, gender, race, etc.) as well as socioeconomic (employment and residential status) factors. Yet, the study did not explore potential behavioral variables such as impulsivity. Impulse inhibition is thought to be central to controlling social behavior (119). This has been corroborated in prior studies such as the one authored by Von Hippel & Gonsalkorale (2005), which revealed that cognitive inhibition predicted more appropriate social behavior [8]. Our study extends this idea to those in recovery, as increased impulsivity is linked to social impairment. Therefore, beyond being a risk factor and a contributor to OUD, impulsivity may also impact patients’ relationships. Since patient-provider relationships and support networks [56] are crucial during treatment, impulsivity may represent a drawback in recovery.

This study adds to this initial finding, exploring the potential mechanisms that underlie this relationship. The added layer of trauma might impact the impulsivity of those in recovery [57]. Peck et al. (2022) found that those with comorbid Post-Traumatic Stress Disorder (PTSD) and OUD were more impulsive in the context of negative emotions compared to those in recovery without PTSD [57]. Furthermore, research has pointed out that experiencing adversity during childhood is linked to poor social outcomes [58] and impulsivity [59, 60]. Aligned with these ideas, our study found that, when controlling for depressive symptoms, the relationship between impulsivity and social functioning is moderated by childhood trauma history.

This finding highlights the complex interplay between these factors. Depressive symptoms symptoms are strongly associated with both childhood trauma and impaired social functioning, potentially masking the moderating effect of ACEs. For instance, depressive symptoms can often manifest as social withdrawal and reduced engagement, which might be misinterpreted as impaired social functioning due to impulsivity or childhood trauma. By controlling for depressive symptoms, we were able to isolate the specific impact of ACEs on the impulsivity-social functioning relationship. This approach revealed that the influence of impulsivity on social functioning varies depending on an individual’s history of childhood adversity, independent of their current depressive state. To our knowledge, this was the first study to find the moderation role of childhood adversity in the context of social functioning and impulsivity for those in recovery. This result suggests that the relationships between trauma, impulsivity, and OUD might be additive.

It is not surprising that individuals with social impairment revealed more significant levels of depression symptoms. Previous studies have found that, in those who have SUD, social impairment is more prominent when it is comorbid with mental health issues [61, 62]. Depressive symptoms were considered at every step of our analysis to explore potential relationships that exist beyond its notorious effects. As discussed, childhood adversity emerged as a moderator only when depression was controlled for, indicating that trauma history affects patients above and beyond the mental health burden alone. Although unexpected, this finding aligns with previous literature that uncovered the broader impact of trauma history on survivors, surpassing mental health concerns [63]. This nuanced understanding is crucial for developing targeted interventions that address the root causes of social impairment in individuals with OUD, rather than focusing solely on depressive symptomatology.

In this study, we explored brain activity during an affective inhibitory task to better understand the neural underpinnings of social functioning. In particular, we aimed to understand if those with social impairment had differential brain activity while engaging in impulse control. While the socially impaired group exhibited higher levels of self-reported impulsivity, there were no differences of performance on the fNIRS Go-No-go task. The lack thereof is not unique to this study; similar results have been reported in other studies employing this paradigm [64–66]. This result could be attributed to the task’s inherent simplicity, corresponding to the observed ceiling effect on performance. Conversely, this facilitated the identification of notable differences in brain activation during comparable behavior.

The SI group revealed greater activation in the dorsolateral (Optodes 1 and 15), ventrolateral (Optode 16), and ventromedial (Optode 10) PFC compared to the N-SI group during both the Go and No-Go blocks of the task. In line with existing literature, differences in neural activation were primarily found in the lateral regions of the PFC [67]. Experiments with clinical populations that exhibit impulse control challenges, such as Attention-Deficit/Hyperactivity Disorder (ADHD) and mania, also revealed activation differences in the ventrolateral [68, 69] and dorsolateral [66] PFC during the Go-No/Go task. The studies, however, reported attenuated activation of these regions for the clinical groups compared to control during impulse inhibition, while in our sample, the socially impaired group displayed increased activation overall. One of the primary differences between this study and the others is the affective nature of our Go/No-Go task, which may elicit greater emotional reactivity in those with social impairment. Given the preliminary nature of these findings and the small sample size, further research is needed to explore whether the increased PFC activation observed here can be replicated and to clarify its functional significance in individuals with substance use disorders.

The PFC has an established role in top-down regulation of behavior, especially in task-relevant responses [70], with some research suggesting that direct stimulation can improve response inhibition [71, 72]. Hence, it would be reasonable to assume that detected heightened activation corresponds to enhanced inhibition. In our sample, however, the increased activation of the PFC did not align with increased performance. One possible explanation for this lies in the suggested role of the PFC in cognitive flexibility. Research suggests that increased PFC activation is associated with adaptive cognitive control [73, 74], helping implement adjustments to adapt to errors or conflicts detected. This could represent a compensatory mechanism, as Weissman et al. (2008) proposed that the role of the PFC in adaptive cognitive control extends to social contexts [75]. Individuals with social impairment may require greater mental effort to adapt their behavior, inhibit responses, and adjust to social norms, possibly explaining the higher PFC activity overall despite similar task performance. However, sample restrictions, including size and gender, also constrain the generalizability of these findings. Future studies are still needed to confirm these patterns and explore the potential neurobiological mechanisms underlying impulse control in individuals with OUD.

### Limitations and future directions

Considering this was a secondary analysis of a modestly sized sample composed of mostly women, it is crucial to replicate these findings with a larger, more balanced sample. As a pilot study, there were challenges with achieving an even distribution of men and women in the sample. Future research should aim for a more gender-balanced sample to better understand how gender differences may influence social functioning in individuals in recovery, as prior studies have suggested that gender can play a significant role in these relationships [4]. Additionally, a case-control study comparing these same relationships in a sample not in recovery from OUD would be beneficial to investigate how social functioning and its associated factors differ between individuals with and individuals without a history of OUD.

Beyond the sample limitations, our study is limited by the complexity of studying social functioning. Due to its relative neglect in research, there are considerable challenges with operationalizing the concept, finding a standardized measure, and establishing an effective methodology for its study. Future research is needed in which participants are recruited based on their social functioning levels while implementing a standardized measure and an experimental approach to evaluate this variable comprehensively. While validated measures were used, the impact of social desirability and self-report items should still be considered. Self-report measures, though valuable, can be subject to biases that may affect data accuracy in this context. Participants may underreport socially undesirable behaviors (e.g., impulsivity, drug use) and overreport desirable ones (e.g., social functioning) due to social desirability bias. The sensitive nature of topics such as substance use, childhood trauma, and social functioning in OUD research makes these potential biases particularly relevant. Future studies could benefit from incorporating multiple assessment methods, such as behavioral tasks or clinician-rated measures, to provide a more comprehensive and potentially less biased assessment of the constructs under study. Finally, despite being a more trauma-informed and community-engaged option for neuroimaging studies, fNIRS restricted our analysis solely to the PFC. Therefore, more investigation is needed, taking advantage of other technologies to analyze these relationships in different brain areas. Despite these limitations, this study is, to our knowledge, the first of its kind to uncover impulsivity and childhood trauma as potential variables underlying the social challenges faced by patients in recovery from OUD.

### Clinical implications

Developing and maintaining relationships is a critical ingredient for the recovery process [31, 76]. Approaches that take advantage of interpersonal relationships, such as peer recovery support [77], have shown great promise in SUD recovery. However, some patients face more social functioning challenges than others, which possibly hampers their recovery process. This study aims to acknowledge and identify areas for potential intervention that could aid in the recovery journey. Our results reveal that, beyond self-reported impulsivity, these individuals exhibit differential brain activation in an inhibitory task, which may help explain the social challenges they are experiencing. Understanding the neural differences in those with and without social functioning challenges can help us destigmatize patients in recovery.

Additionally, as underscored by Van Reekum et al. (2020), there is an evident gap in addressing the social needs of patients with OUD during recovery [4]. Impulsivity, along with corresponding neural alterations, may, hence, represent a promising area for treatment development. Considering the impact of impulsivity on social impairment varies depending on the history of childhood trauma, it is crucial to implement a trauma-informed, holistic, transdiagnostic approach to OUD recovery. This was particularly true when looking beyond the impact of mental health symptoms, which highlights the importance of, in addition to mental health evaluations, implementing trauma history screening before, and throughout the development of the recovery care plan. This could help generate more personalized and productive strategies to support the social functioning needs of those in recovery.

## Conclusion

Social impairment is a hallmark of OUD, and it can severely impact recovery. This study revealed how social challenges are related to impulsivity and that experiencing childhood trauma can further exacerbate this relationship. Although a notable outcome of OUD, depression symptoms can confound relationships that exist beyond its burden, making it crucial for other variables (such as trauma and impulsivity) to be evaluated and accounted for. By understanding what lies behind the social challenges patients face in recovery, we can implement novel targets that maximize social adjustment during and after recovery.

## Data Availability

The datasets used and/or analyzed during the current study are available from the corresponding author on reasonable request.

## Abbreviations

OUD: Opioid use disorder
PFC: Prefrontal cortex
ACEs: Adverse Childhood Experiences

## References

[1] Opioid overdose [Internet]. [cited 2024 Mar 24]. Available from: https://www.who.int/news-room/fact-sheets/detail/opioid-overdose

[2] Trujols J, Garijo I, Siñol N, Del Pozo J, Portella MJ. Pérez De Los Cobos J. Patient satisfaction with methadone maintenance treatment: the relevance of participation in treatment and social functioning. Drug Alcohol Depend. 2012;123(1–3):41–7. 22071121 10.1016/j.drugalcdep.2011.10.014

[3] Ashrafioun L, Allan NP, Stecker TA. Opioid use disorder and its association with self-reported difficulties participating in social activities. Am J Addict. 2022;31(1):46–52. 34472669 10.1111/ajad.13220

[4] Van Reekum EA, Rosic T, Hudson J, Sanger N, Marsh DC, Worster A, et al. Social functioning outcomes in men and women receiving medication-assisted treatment for opioid use disorder. Biol Sex Differ. 2020;11(1):20. 32326982 10.1186/s13293-020-00298-4 PMC7181574

[5] McDonald S, Darke S, Kaye S, Torok M. Deficits in social perception in opioid maintenance patients, abstinent opioid users and non-opioid users. Addiction. 2013;108(3):566–74. 23164063 10.1111/add.12040

[6] Bandura A. Social cognitive theory of self-regulation. Organ Behav Hum Decis Process. 1991;50(2):248–87.

[7] Von Hippel W, Gonsalkorale K. That is bloody revolting! Inhibitory Control of Thoughts Better Left Unsaid. Psychol Sci. 2005;16(7):497–500. 16008778 10.1111/j.0956-7976.2005.01563.x

[8] Stanford MS, Greve KW, Boudreaux JK, Mathias CW, Brumbelow L. Impulsiveness and risk-taking behavior: comparison of high-school and college students using the Barratt Impulsiveness Scale. Pers Indiv Differ. 1996;21(6):1073–5.

[9] Soni U, Sharma R, Sharma M, Khurana E, Chopra J, Julka D et al. Impulsivity and risk-taking behavior in school-going adolescents. Cureus [Internet]. 2023 Jun 21 [cited 2023 Dec 11]. Available from: https://www.cureus.com/articles/158130-impulsivity-and-risk-taking-behavior-in-school-going-adolescents 10.7759/cureus.40728 PMC10360448 37485185

[10] Evren C, Bozkurt M. Impulsivity and opioid use disorder. Dusunen Adam J Psychiatry Neurol Sci. 2017;30(2):75–8. 10.5350/DAJPN20173002001.

[11] Vest N, Reynolds CJ, Tragesser SL. Impulsivity and risk for prescription opioid misuse in a chronic pain patient sample. Addict Behav. 2016;60:184–90. 27156219 10.1016/j.addbeh.2016.04.015

[12] Aron AR. The neural basis of inhibition in cognitive control. Neuroscientist. 2007;13(3):214–28. 17519365 10.1177/1073858407299288

[13] Seney ML, Kim SM, Glausier JR, Hildebrand MA, Xue X, Zong W, et al. Transcriptional alterations in Dorsolateral Prefrontal Cortex and nucleus accumbens implicate neuroinflammation and synaptic remodeling in Opioid Use Disorder. Biol Psychiatry. 2021;90(8):550–62. 34380600 10.1016/j.biopsych.2021.06.007 PMC8463497

[14] Lee YK, Gold MS, Fuehrlein BS. Looking beyond the opioid receptor: a desperate need for new treatments for opioid use disorder. J Neurol Sci. 2022;432:120094. 34933249 10.1016/j.jns.2021.120094

[15] Qiu Y, wei, Jiang G hua, Su Hhuan, Lv X, fei, Tian J, zhang, Li L et al. ming,. The impulsivity behavior is correlated with prefrontal cortex gray matter volume reduction in heroin-dependent individuals. Neurosci Lett. 2013;538:43–8. 10.1016/j.neulet.2013.01.019 23352851

[16] Lee TMC, Zhou W, hua, Luo X, jing, Yuen KSL, Ruan X, zhong. Weng X Chu. Neural activity associated with cognitive regulation in heroin users: a fMRI study. Neurosci Lett. 2005;382(3):211–6. 15925092 10.1016/j.neulet.2005.03.053

[17] Ceceli AO, King SG, McClain N, Alia-Klein N, Goldstein RZ. The neural signature of impaired Inhibitory Control in individuals with Heroin Use Disorder. J Neurosci. 2023;43(1):173–82. 36396402 10.1523/JNEUROSCI.1237-22.2022 PMC9838696

[18] Bjorklund DF, Harnishfeger KK. The evolution of inhibition mechanisms and their role in human cognition and behavior. In: Interference and Inhibition in cognition [Internet]. Elsevier; 1995 [cited 2023 Dec 5]. pp. 141–73. Available from: https://linkinghub.elsevier.com/retrieve/pii/B9780122089305500064

[19] Mah L, Arnold MC, Grafman J. Impairment of social perception associated with lesions of the prefrontal cortex. AJP. 2004;161(7):1247–55. 10.1176/appi.ajp.161.7.1247 15229058

[20] Bramham J, Morris RG, Hornak J, Bullock P, Polkey CE. Social and emotional functioning following bilateral and unilateral neurosurgical prefrontal cortex lesions. J Neuropsychol. 2009;3(1):125–43. 19338721 10.1348/174866408X293994

[21] Anderson SW, Bechara A, Damasio H, Tranel D, Damasio AR. Impairment of social and moral behavior related to early damage in human prefrontal cortex. Nat Neurosci. 1999;2(11):1032–7. 10526345 10.1038/14833

[22] Bicks LK, Koike H, Akbarian S, Morishita H. Prefrontal cortex and social cognition in mouse and man. Front Psychol [Internet]. 2015 Nov 26 [cited 2023 Oct 4];6. Available from: 10.3389/fpsyg.2015.01805/abstract 10.3389/fpsyg.2015.01805 PMC4659895 26635701

[23] Santo T, Campbell G, Gisev N, Tran LT, Colledge S, Di Tanna GL, et al. Prevalence of childhood maltreatment among people with opioid use disorder: a systematic review and meta-analysis. Drug Alcohol Depend. 2021;219:108459. 33401031 10.1016/j.drugalcdep.2020.108459 PMC7855829

[24] Teicher MH, Samson JA, Anderson CM, Ohashi K. The effects of childhood maltreatment on brain structure, function and connectivity. Nat Rev Neurosci. 2016;17(10):652–66. 27640984 10.1038/nrn.2016.111

[25] Hogarth L, Martin L, Seedat S. Relationship between childhood abuse and substance misuse problems is mediated by substance use coping motives, in school attending South African adolescents. Drug Alcohol Depend. 2019;194:69–74. 30412899 10.1016/j.drugalcdep.2018.10.009 PMC6327152

[26] Marshall DF, Passarotti AM, Ryan KA, Kamali M, Saunders EFH, Pester B, et al. Deficient inhibitory control as an outcome of childhood trauma. Psychiatry Res. 2016;235:7–12. 26707783 10.1016/j.psychres.2015.12.013 PMC6639093

[27] Dixon KE, Bindbeutel KM, Daugherty YT, Robertson AC, Lee M, Galano MM et al. The differential impact of childhood trauma on adult impulsivity and impulse control. Traumatology [Internet]. 2023 Mar 13 [cited 2023 Dec 11]. Available from: http://doi.apa.org/getdoi.cfm?doi=10.1037/trm0000440

[28] Raposa EB, Hammen C. A daily diary investigation of the influence of early family adversity on social functioning during the transition to adulthood. Soc Dev. 2018;27(2):431–46. 30034119 10.1111/sode.12269 PMC6051721

[29] Aditi B, Arunjyoti B. Addiction severity, social functioning, and life satisfaction of patients diagnosed with substance use disorders. Indian J Psy Nsg. 2018;15(2):13.

[30] Pettersen H, Landheim A, Skeie I, Biong S, Brodahl M, Oute J, et al. How Social relationships Influence Substance Use Disorder Recovery: a collaborative narrative study. Subst Abuse. 2019;13:117822181983337. 10.1177/1178221819833379 PMC6410387 30886519

[31] Moore TM, Reise SP, Gur RE, Hakonarson H, Gur RC. Psychometric properties of the Penn Computerized Neurocognitive Battery. Neuropsychology. 2015;29(2):235–46. 25180981 10.1037/neu0000093 PMC4345134

[32] Bode RK, Hahn EA, DeVellis R, Cella D. Measuring participation: the patient-reported outcomes Measurement Information System Experience. Arch Phys Med Rehabil. 2010;91(9):S60–5. 20801282 10.1016/j.apmr.2009.10.035 PMC3671872

[33] Rothrock NE, Amtmann D, Cook KF. Development and validation of an interpretive guide for PROMIS scores. J Patient Rep Outcomes. 2020;4(1):16. 32112189 10.1186/s41687-020-0181-7 PMC7048882

[34] Reise SP, Moore TM, Sabb FW, Brown AK, London ED. The Barratt Impulsiveness Scale–11: reassessment of its structure in a community sample. Psychol Assess. 2013;25(2):631–42. 23544402 10.1037/a0032161 PMC3805371

[35] Vasconcelos AG, Malloy-Diniz L, Correa H. Systematic review of psychometric proprieties of Barratt impulsiveness scale version 11 (BIS-11). Clin Neuropsychiatry. 2012;9(2).

[36] Cronholm PF, Forke CM, Wade R, Bair-Merritt MH, Davis M, Harkins-Schwarz M, et al. Adverse childhood experiences. Am J Prev Med. 2015;49(3):354–61. 26296440 10.1016/j.amepre.2015.02.001

[37] Alhowaymel FM, Kalmakis KA, Chiodo LM, Kent NM, Almuneef M. Adverse childhood experiences and Chronic diseases: identifying a cut-point for ACE scores. IJERPH. 2023;20(2):1651. 36674405 10.3390/ijerph20021651 PMC9863315

[38] Lacey RE, Minnis H. Practitioner review: twenty years of research with adverse childhood experience scores– advantages, disadvantages and applications to practice. Child Psychol Psychiatry. 2020;61(2):116–30. 10.1111/jcpp.13135 31609471

[39] Rush AJ, Trivedi MH, Ibrahim HM, Carmody TJ, Arnow B, Klein DN, et al. The 16-Item quick inventory of depressive symptomatology (QIDS), clinician rating (QIDS-C), and self-report (QIDS-SR): a psychometric evaluation in patients with chronic major depression. Biol Psychiatry. 2003;54(5):573–83. 12946886 10.1016/s0006-3223(02)01866-8

[40] Trivedi MH, Rush AJ, Ibrahim HM, Carmody TJ, Biggs MM, Suppes T, et al. The Inventory of Depressive Symptomatology, Clinician Rating (IDS-C) and self-report (IDS-SR), and the Quick Inventory of Depressive Symptomatology, Clinician Rating (QIDS-C) and self-report (QIDS-SR) in public sector patients with mood disorders: a psychometric evaluation. Psychol Med. 2004;34(1):73–82. 14971628 10.1017/s0033291703001107

[41] Ayaz H, Baker WB, Blaney G, Boas DA, Bortfeld H, Brady K et al. Optical imaging and spectroscopy for the study of the human brain: status report. Neurophoton [Internet]. 2022 Aug 30 [cited 2024 Mar 31];9(S2). Available from: https://www.spiedigitallibrary.org/journals/neurophotonics/volume-9/issue-S2/S24001/Optical-imaging-and-spectroscopy-for-the-study-of-the-human/10.1117/1.NPh.9.S2.S24001.full 10.1117/1.NPh.9.S2.S24001 PMC9424749 36052058

[42] Cutini S, Brigadoi S. Unleashing the future potential of functional near-infrared spectroscopy in brain sciences. J Neurosci Methods. 2014;232:152–6. 24880046 10.1016/j.jneumeth.2014.05.024

[43] Herold F, Wiegel P, Scholkmann F, Müller N. Applications of functional near-infrared spectroscopy (fNIRS) neuroimaging in exercise–cognition science: a systematic, methodology-focused review. JCM. 2018;7(12):466. 30469482 10.3390/jcm7120466 PMC6306799

[44] Curtin A, Ayaz H. The age of neuroergonomics: towards ubiquitous and continuous measurement of brain function with fNIRS. Jpn Psychol Res. 2018;60(4):374–86.

[45] Ayaz H, Shewokis PA, Curtin A, Izzetoglu M, Izzetoglu K, Onaral B. Using MazeSuite and Functional Near Infrared Spectroscopy to Study Learning in spatial Navigation. JoVE. 2011;56:3443. 10.3791/3443 PMC3227178 22005455

[46] Ayaz H, Shewokis PA, Bunce S, Izzetoglu K, Willems B, Onaral B. Optical brain monitoring for operator training and mental workload assessment. NeuroImage. 2012;59(1):36–47. 21722738 10.1016/j.neuroimage.2011.06.023

[47] Goldman M, Ehrman RN, Suh JJ, Hurt H, Marquez K, Franklin TR, et al. Brief report: Spiders-No, Puppies‐Go, introducing a novel go NoGo task tested in inner city adolescents at risk for poor impulse control. J Adolesc. 2015;38(1):45–8. 25460679 10.1016/j.adolescence.2014.10.007 PMC4268313

[48] Peirce J, Gray JR, Simpson S, MacAskill M, Höchenberger R, Sogo H, et al. PsychoPy2: experiments in behavior made easy. Behav Res. 2019;51(1):195–203. 10.3758/s13428-018-01193-y PMC6420413 30734206

[49] Fishburn FA, Ludlum RS, Vaidya CJ, Medvedev AV. Temporal derivative distribution repair (TDDR): a motion correction method for fNIRS. NeuroImage. 2019;184:171–9. 30217544 10.1016/j.neuroimage.2018.09.025 PMC6230489

[50] Tormohlen KN, Mojtabai R, Seiwell A, McGinty EE, Stuart EA, Tobin KE, et al. Co-occurring opioid Use and Depressive disorders: patient characteristics and Co-occurring Health conditions. J Dual Diagnosis. 2021;17(4):296–303. 10.1080/15504263.2021.1979349 PMC10294295 34581663

[51] Bernet CZ, Stein MB. Relationship of childhood maltreatment to the onset and course of major depression in adulthood. Depress Anxiety. 1999;9(4):169–74. 10431682

[52] Youngren MA, Lewinsohn PM. The functional relation between depression and problematic interpersonal behavior. J Abnorm Psychol. 1980;89(3):333–41. 7410700 10.1037//0021-843x.89.3.333

[53] Rodríguez-Cintas L, Daigre C, Grau-López L, Barral C, Pérez-Pazos J, Voltes N, Braquehais MD, Casas M, Roncero C. Impulsivity and addiction severity in cocaine and opioid dependent patients. Addict Behav. 2016;58:104–9. 10.1016/j.addbeh.2016.02.029. 26922157 10.1016/j.addbeh.2016.02.029

[54] Poling J, Kosten TR, Sofuoglu M. Treatment outcome predictors for cocaine dependence. Am J Drug Alcohol Abus. 2007;33(2):191–206. 10.1080/00952990701199416. 10.1080/00952990701199416 17497542

[55] Oles W, Alexander M, Kumar N, Howell B, O’Connor PG, Madden LM, et al. Characterizing the social support and functioning of a low-threshold medication for opioid use disorder treatment cohort at intake. BMC Psychiatry. 2022;22(1):236. 35366844 10.1186/s12888-022-03884-5 PMC8976510

[56] Kumar N, Oles W, Howell BA, Janmohamed K, Lee ST, Funaro MC, et al. The role of social network support in treatment outcomes for medication for opioid use disorder: a systematic review. J Subst Abuse Treat. 2021;127:108367. 34134871 10.1016/j.jsat.2021.108367 PMC9022048

[57] Peck KR, Nighbor TD, Price M. Examining associations between impulsivity, opioid use disorder, and posttraumatic stress disorder: the additive relation between disorders. Exp Clin Psychopharmacol. 2022;30(5):486–93. 34291989 10.1037/pha0000507 PMC8782919

[58] Haahr-Pedersen I, Perera C, Hyland P, Vallières F, Murphy D, Hansen M, et al. Females have more complex patterns of childhood adversity: implications for mental, social, and emotional outcomes in adulthood. Eur J Psychotraumatology. 2020;11(1):1708618. 10.1080/20008198.2019.1708618 PMC6968572 32002142

[59] Lovallo WR. Early life adversity reduces stress reactivity and enhances impulsive behavior: implications for health behaviors. Int J Psychophysiol. 2013;90(1):8–16. 23085387 10.1016/j.ijpsycho.2012.10.006 PMC3587283

[60] Mackey S, Chaarani B, Kan KJ, Spechler PA, Orr C, Banaschewski T, et al. Brain regions related to Impulsivity mediate the effects of early adversity on antisocial behavior. Biol Psychiatry. 2017;82(4):275–82. 26971049 10.1016/j.biopsych.2015.12.027

[61] Menezes PR, Johnson S, Thornicroft G, Marshall J, Prosser D, Bebbington P, et al. Drug and alcohol problems among individuals with Severe Mental Illnesses in South London. Br J Psychiatry. 1996;168(5):612–9. 8733801 10.1192/bjp.168.5.612

[62] Link BG, Struening EL, Rahav M, Phelan JC, Nuttbrock L. On stigma and its consequences: evidence from a longitudinal study of men with dual diagnoses of mental illness and substance abuse. J Health Soc Behav. 1997;38(2):177–90. 9212538

[63] Sinko L, Ploutz-Snyder R, Kramer MM, Conley T, Arnault DS. Trauma history as a significant predictor of Posttraumatic Growth Beyond Mental Health Symptoms in women-identifying survivors of undergraduate non-consensual sexual experiences. Violence Vict. 2022;37(3):396–421. 35654488 10.1891/VV-D-20-00082

[64] Miao S, Han J, Gu Y, Wang X, Song W, Li D, et al. Reduced Prefrontal Cortex activation in children with Attention-Deficit/Hyperactivity disorder during Go/No-Go Task: a functional Near-Infrared Spectroscopy Study. Front Neurosci. 2017;11:367. 28701914 10.3389/fnins.2017.00367 PMC5487426

[65] Sinko L, Regier P, Curtin A, Ayaz H, Rose Childress A, Teitelman AM. Neural correlates of cognitive control in women with a history of sexual violence suggest altered prefrontal cortical activity during cognitive processing. Womens Health (Lond Engl). 2022;18:174550572210813. 10.1177/17455057221081326 PMC8883288 35225075

[66] Wu T, Liu X, Cheng F, Wang S, Li C, Zhou D, et al. Dorsolateral prefrontal cortex dysfunction caused by a go/no-go task in children with attention-deficit hyperactivity disorder: a functional near-infrared spectroscopy study. Front Neurosci. 2023;17:1145485. 37056303 10.3389/fnins.2023.1145485 PMC10086251

[67] Rodrigo AH, Di Domenico SI, Graves B, Lam J, Ayaz H, Bagby RM, et al. Linking trait-based phenotypes to prefrontal cortex activation during inhibitory control. Soc Cogn Affect Neurosci. 2016;11(1):55–65. 26163672 10.1093/scan/nsv091 PMC4692316

[68] Elliott R, Ogilvie A, Rubinsztein JS, Calderon G, Dolan RJ, Sahakian BJ. Abnormal ventral frontal response during performance of an affective go/no go task in patients with mania. Biol Psychiatry. 2004;55(12):1163–70. 15184035 10.1016/j.biopsych.2004.03.007

[69] Mazzola-Pomietto P, Kaladjian A, Azorin JM, Anton JL, Jeanningros R. Bilateral decrease in ventrolateral prefrontal cortex activation during motor response inhibition in mania. J Psychiatr Res. 2009;43(4):432–41. 18586275 10.1016/j.jpsychires.2008.05.004

[70] Brosnan MB, Wiegand I. The dorsolateral prefrontal cortex, a dynamic cortical area to enhance top-down attentional control. J Neuroscience Off J Soc Neurosci. 2017;37(13):3445–6. 10.1523/JNEUROSCI.0136-17.2017. 10.1523/JNEUROSCI.0136-17.2017 PMC6596925 28356395

[71] Boggio PS, Bermpohl F, Vergara AO, Muniz ALCR, Nahas FH, Leme PB, et al. Go-no-go task performance improvement after anodal transcranial DC stimulation of the left dorsolateral prefrontal cortex in major depression. J Affect Disord. 2007;101(1–3):91–8. 17166593 10.1016/j.jad.2006.10.026

[72] Chen T, Wang H, Wang X, Zhu C, Zhang L, Wang K, et al. Transcranial direct current stimulation of the right dorsolateral prefrontal cortex improves response inhibition. Int J Psychophysiol. 2021;162:34–9. 33497765 10.1016/j.ijpsycho.2021.01.014

[73] Egner T, Hirsch J. Cognitive control mechanisms resolve conflict through cortical amplification of task-relevant information. Nat Neurosci. 2005;8(12):1784–90. 16286928 10.1038/nn1594

[74] Gbadeyan O, McMahon K, Steinhauser M, Meinzer M. Stimulation of Dorsolateral Prefrontal Cortex enhances adaptive cognitive control: a high-definition Transcranial Direct Current Stimulation Study. J Neurosci. 2016;36(50):12530–6. 27974612 10.1523/JNEUROSCI.2450-16.2016 PMC6705663

[75] Weissman DH, Perkins AS, Woldorff MG. Cognitive control in social situations: a role for the dorsolateral prefrontal cortex. NeuroImage. 2008;40(2):955–62. 18234518 10.1016/j.neuroimage.2007.12.021 PMC2522237

[76] Moos RH. Theory-based active ingredients of effective treatments for substance use disorders. Drug Alcohol Depend. 2007;88(2–3):109–21. 17129682 10.1016/j.drugalcdep.2006.10.010 PMC1896183

[77] Reif S, Braude L, Lyman DR, Dougherty RH, Daniels AS, Ghose SS, et al. Peer recovery support for individuals with Substance Use disorders: assessing the evidence. PS. 2014;65(7):853–61. 10.1176/appi.ps.201400047 24838535

